# Age-Related Sexual Dimorphism in Temporal Discrimination and in Adult-Onset Dystonia Suggests GABAergic Mechanisms

**DOI:** 10.3389/fneur.2015.00258

**Published:** 2015-12-14

**Authors:** John S. Butler, Ines M. Beiser, Laura Williams, Eavan McGovern, Fiona Molloy, Tim Lynch, Dan G. Healy, Helena Moore, Richard Walsh, Richard B. Reilly, Seán O’Riordan, Cathal Walsh, Michael Hutchinson

**Affiliations:** ^1^Trinity Centre for Bioengineering, School of Engineering, Trinity College Dublin, Dublin, Ireland; ^2^Department of Neurology, St. Vincent’s University Hospital, Dublin, Ireland; ^3^School of Medicine and Medical Sciences, University College Dublin, Dublin, Ireland; ^4^Beaumont Hospital, Dublin, Ireland; ^5^Dublin Neurological Institute, Mater Misericordiae University Hospital, Dublin, Ireland; ^6^Cork University Hospital, Cork, Ireland; ^7^Adelaide and Meath Hospital, Dublin, Ireland; ^8^School of Medicine, Trinity College Dublin, Dublin, Ireland; ^9^Department of Statistics, Trinity College Dublin, Dublin, Ireland; ^10^Department of Mathematics and Statistics, University of Limerick, Limerick, Ireland

**Keywords:** adult-onset isolated focal dystonia, sex ratio, temporal discrimination, sexual dimorphism, penetrance

## Abstract

**Background:**

Adult-onset isolated focal dystonia (AOIFD) presenting in early adult life is more frequent in men, whereas in middle age it is female predominant. Temporal discrimination, an endophenotype of adult-onset idiopathic isolated focal dystonia, shows evidence of sexual dimorphism in healthy participants.

**Objectives:**

We assessed the distinctive features of age-related sexual dimorphism of (i) sex ratios in dystonia phenotypes and (ii) sexual dimorphism in temporal discrimination in unaffected relatives of cervical dystonia patients.

**Methods:**

We performed (i) a meta-regression analysis of the proportion of men in published cohorts of phenotypes of adult-onset dystonia in relation to their mean age of onset and (ii) an analysis of temporal discrimination thresholds in 220 unaffected first-degree relatives (125 women) of cervical dystonia patients.

**Results:**

In 53 studies of dystonia phenotypes, the proportion of men showed a highly significant negative association with mean age of onset (*p* < 0.0001, pseudo-*R*^2^ = 59.6%), with increasing female predominance from 40 years of age. Age of onset and phenotype together explained 92.8% of the variance in proportion of men. Temporal discrimination in relatives under the age of 35 years is faster in women than men but the age-related rate of deterioration in women is twice that of men; after 45 years of age, men have faster temporal discrimination than women.

**Conclusion:**

Temporal discrimination in unaffected relatives of cervical dystonia patients and sex ratios in adult-onset dystonia phenotypes show similar patterns of age-related sexual dimorphism. Such age-related sexual dimorphism in temporal discrimination and adult-onset focal dystonia may reflect common underlying mechanisms. Cerebral GABA levels have been reported to show similar age-related sexual dimorphism in healthy participants and may be the mechanism underlying the observed age-related sexual dimorphism in temporal discrimination and the sex ratios in AOIFD.

## Introduction

Twenty years ago, a paper from the late David Marsden’s research group, reporting on the sex ratios of the phenotypes of adult-onset dystonia, concluded by stating: “*Our study confirms a clear but mild preponderance of females with various types of craniocervical dystonia and of males with writer’s cramp. Why this is so remains to be discovered*” (1).

Dystonia is a movement disorder, characterized by “sustained or intermittent muscle contractions causing abnormal, often repetitive, movements, postures, or both” (2). The most common form of dystonia, adult-onset isolated focal dystonia (AOIFD) is inherited in an autosomal dominant manner with a reduced penetrance of 12–15% (3, 4); phenotypes include cervical dystonia, blepharospasm, focal hand dystonia, spasmodic dysphonia, oromandibular dystonia, and task-specific dystonia. Evidence from studies of affected sib-pairs (5) and multiplex families (6, 7) indicates that the same presumed genetic mutation(s) may cause different phenotypes. There is an unexplained male predominance in focal hand dystonia and musician’s dystonia with a female excess in the craniocervical phenotypes.

The temporal discrimination threshold (TDT) is the shortest interval at which two sequential stimuli appear to the observer to be asynchronous; normally, the TDT is 30–50 ms depending on the gender and age (8). Abnormal TDTs are found in 97% of cervical dystonia patients (specificity: 98–100%) and somewhat less frequently in other AOIFD phenotypes (8–10). Abnormal TDTs show autosomal dominant transmission in families of sporadic and familial cervical dystonia patients (8, 11). In unaffected first-degree female relatives, an abnormal TDT is fully penetrant by 48 years of age; in male relatives, there is 40% penetrance after 25 years of age (12). Abnormal TDTs in unaffected first-degree relatives of patients with AOIFD are associated with putaminal hypertrophy (8) and reduced putaminal fMRI activity during a temporal discrimination task (12). We have proposed that an abnormal TDT is a mediational endophenotype of AOIFD and that unaffected first-degree relatives (of AOIFD patients) with abnormal TDTs are unaffected gene carriers (13, 14).

In a group of healthy control participants, we observed that young women had faster temporal discrimination than similarly aged men (12). Further analysis indicated both sex-related and age-related effects on the TDT. TDTs worsened with age at a faster rate in women than men, so that in middle age the initial female advantage was lost; women after 40 years were significantly slower in a temporal discrimination task than men (15).

Sexual dimorphism, a phenotypic difference between males and females of the same species, in temporal discrimination is age dependent; the initial advantage of the young adult woman is lost and reversed in middle age. In this paper, we further examine this sexual dimorphism in temporal discrimination in a large group of unaffected first-degree relatives of cervical dystonia patients. We also examine by meta-analysis the relationship between mean age of onset and sex ratios in published cohorts of AOIFD phenotypes. We postulate that the age-related sexual dimorphism in temporal discrimination explains the relationship between and the mean age of onset and sex ratios of AOIFD phenotypes in both published clinical cohorts and in our cervical dystonia patients. We hypothesize that, in individuals with a genetic susceptibility to AOIFD, age- and sex-related mechanisms relating to the efficiency of temporal discrimination explain the observed sex ratios in the various AOIFD phenotypes. One further corollary hypothesis, discussed but not examined in this paper, is that sexual dimorphism in the speed of temporal discrimination reflects sexual dimorphism in physiological age-related decline in GABAergic inhibition.

## Participants and Methods

### Study A: Age of Onset, Phenotypes, and Sex Ratios in AOIFD

#### Study A Population

We searched, in January 2015, PubMed and MEDLINE for publications using the search terms “adult-onset dystonia,” “focal dystonia,” “blepharospasm,” “cervical dystonia,” focal hand dystonia,” “writer’s cramp,” “spasmodic dysphonia,” “laryngeal dystonia,” oromandibular dystonia,” “Meige syndrome,” “muscician’s dystonia,” “sex ratio,” “dystonia,” “epidemiology,” “incidence,” and “prevalence.” Our inclusion criteria were publications that reported the numbers of patients, sex ratios (proportion of men), and mean age of onset in clearly defined AOIFD phenotypes from a clinic or study sample population. Three authors (Ines M. Beiser, Michael Hutchinson, and Seán O’Riordan) screened full texts to identify study eligibility. We included only studies that were published in English; the references of all eligible studies were searched to ensure that no study was missed. Exclusion criteria were studies which did not separate secondary dystonias or dystonia-plus syndromes and repeated studies from the same geographical region (in which case the largest or most recent study was used). A table of included studies, listed by phenotype and references, is given in Table S1 in Supplementary Material.

#### Statistical Analysis

A mixed effects meta-regression model was fitted to the logit of the sex ratio (proportion of cases that were male to total number of cases) with (i) mean age of onset and (ii) mean age of onset plus phenotype being included as moderators in the model. The model was fitted in R version 3.1.1 using the package metafor version 1.9.3 (16). In reporting, the model’s estimates of the effect sizes and SEs, together with pseudo-*R*^2^, defined as $\left(\tau_{\text{RE}}^{2}-\tau_{\text{ME}}^{2}\right)/\tau_{\text{RE}}^{2}$ are presented (17).

### Study B: Age- and Sex-Related Effects on Temporal Discrimination in Relatives of Cervical Dystonia Patients

#### Participants

Two hundred twenty unaffected first-degree relatives of cervical dystonia patients between the ages of 18 and 65 years (125 women, mean age 40.2 years; 95 men, mean age 39.7 years) were recruited by initial contact with cervical dystonia patients and subsequently gave full informed consent. A full medical history was taken, and the relatives were assessed for any evidence of a neurological disorder. Exclusion criteria were a history of neurological disease, including dystonia, tremor, neuropathy, visual or cognitive impairment, a history of cerebral, and cervical or brachial plexus injury. The TDT results from 175 healthy control participants (88 women, mean age 40.5 years; 87 men, mean age 21.5 years) from a previous study were included as a control group (15).

#### Methods: Sensory Testing: Visual and Tactile TDT Testing

Methods: sensory testing: visual and tactile TDT testing was performed in a single session; in a soundproof, darkened room as described previously. Visual stimuli (two flashing LED lights) were positioned on a table, 7° in the participant’s periphery. Tactile stimuli (non-painful electrical impulses to the index and middle fingers) were presented using square-wave stimulators (Lafayette Instruments Europe, United Kingdom) and rectangular cloth electrodes (Item # TD-141C1, Discount Disposables, St. Albans, VT, USA). The stimulus current was manually increased (in 0.1 mA steps) until the participant could reliably detect the stimuli. Visual or tactile stimuli, 5 ms in duration, were presented at 5-s intervals. The stimuli were initially synchronous and separation between pairs of stimuli was introduced in 5 ms steps. When the participant reported stimuli to be asynchronous on three consecutive occasions, the first of these was taken as the TDT. Visual and tactile testing was repeated four times on each side of the body (a total of 16 runs) in a random order and the median (milliseconds) of the four trials was used to account for a practice effect. Means of the median visual, tactile, and combined values were calculated (TDT). Testing was carried out by the research registrars according to a standardized protocol.

#### Statistical Analysis

To investigate the effect of age and sex on temporal discrimination, regression analyses were performed. The combined TDTs for men and for women were submitted to regression analyses with age as the continuous variable. The *F* values, *R*^2^ values, and corresponding *p* values are reported along with 95% confidence intervals, *t*-values, and *p* values for the intercept; and β value for the linear fit. To compare the intercept and β values between men and women, a regression analysis was performed on the TDT data with variables age, sex (men = 0, women = 1) and age × sex (resulting in 0 s for men and the continuous variable of age for women). The sex variable tests for differences in the intercept values between relatives and women. The age × sex variable tests for differences in the β values between men and women. To investigate intercept and β values differences between relatives and control participants, a regression analysis was performed on the TDT data with variables age, group (relatives = 0, controls = 1) and age × sex (resulting in 0 s for relatives and the continuous variable of age for controls). The group variable tests for differences in the intercept values between relatives and controls. The age × group variable tests for differences in the β values between relatives and controls. Approval for this project was obtained from the Ethics and Medical Research Committee, St. Vincent’s University Hospital.

### Study C: Cervical Dystonia: Difference in Mean Age of Onset by Sex

From our clinic population of 331 cervical dystonia patients, the age of onset could be reliably determined by report in 278 patients (188 women). Three other studies, which reported mean age of onset of cervical dystonia for both sexes, were also used in a meta-analysis of mean age of onset by sex in cervical dystonia (16–18).

#### Statistical Analysis

Meta-analysis of the mean age of onset by sex in our cohort and three other studies (18–20), which reported mean age of onset of cervical dystonia for both sexes, was performed and the mean difference in age of onset between men and women presenting with cervical dystonia was determined. A random effects meta-analysis was performed using Review Manager version 5.3, Cochrane database tool (http://tech.cochrane.org/revman).

## Results

### Study A: Age of Onset, Phenotypes, and Sex Ratios in AOIFD

#### Analysis Set

A total of 78 papers fulfilling the search criteria were found. Of these, 24 studies listed both the mean age of onset and the sex ratio for the AOIFD phenotypes of interest; there were 54 papers which did not provide adequate information for analysis or were repeated studies from the same site/region. The 24 included studies generated a total of 53 reports of mean age of onset and sex ratio for the each of the five phenotypes: cervical dystonia (15 reports), focal hand dystonia (12 reports), musician’s dystonia (3 reports), laryngeal dystonia (9 reports), and blepharospasm (14 reports). A list of all analyzed studies and phenotypes is available in Table S1 in Supplementary Material.

#### Meta-Regression Analysis of Relationship Between Sex Ratio and Mean Age of Onset

**Figure 1:**
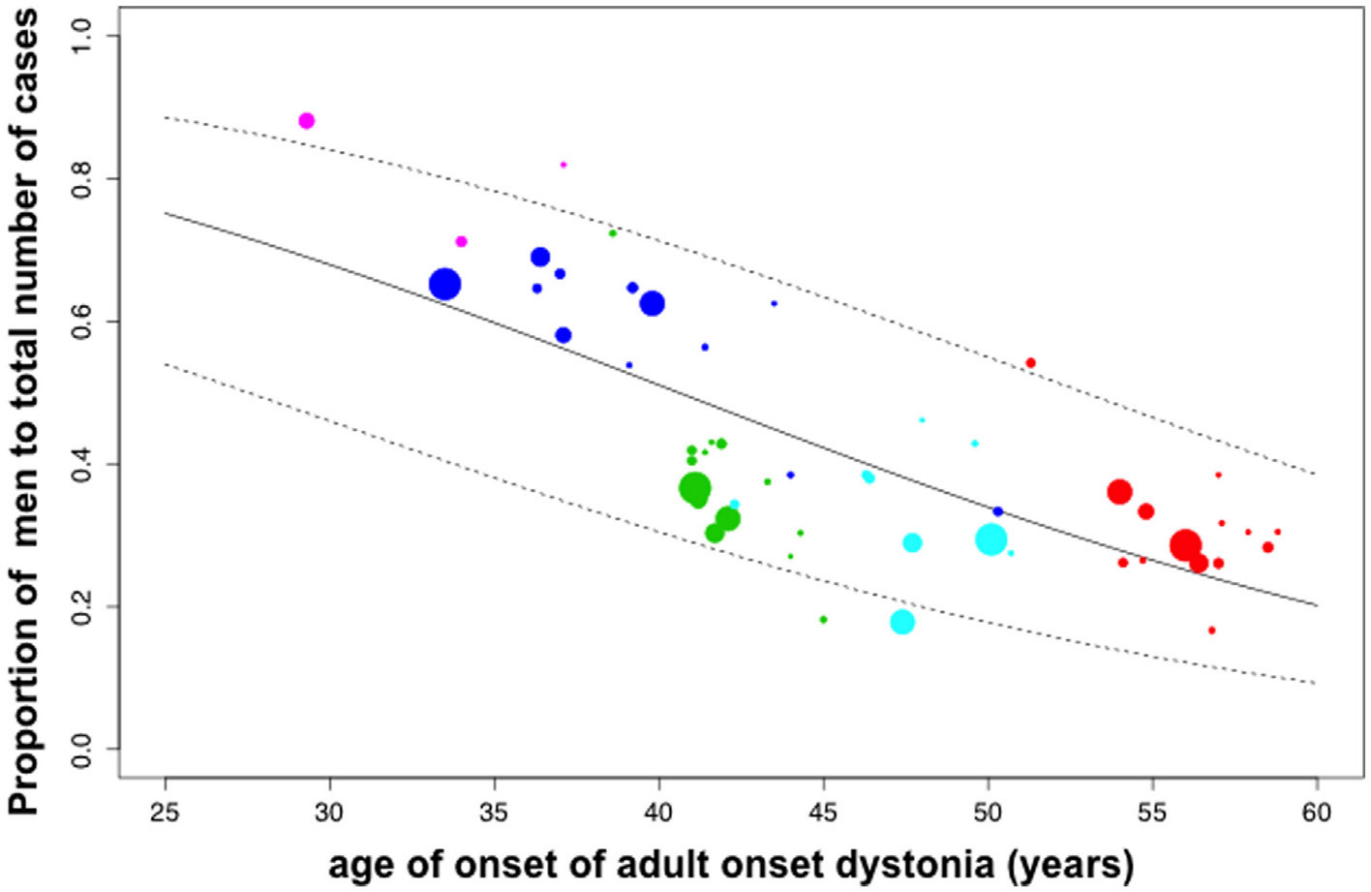
**Meta-regression analysis of the relationship between sex ratio (proportion of men) and mean age of onset in phenotypes of adult-onset dystonia**. Fifty-three reports of mean age of onset and sex ratio of five adult-onset dystonia phenotypes (listed in Table S1 in Supplementary Material). In each filled circle, the diameter of the circle is proportional to the square root of the number of individuals in that study. Circle color indicates musician’s dystonia (pink), focal hand dystonia (blue), cervical dystonia (green), laryngeal dystonia (turquoise), and blepharospasm (red). A linear decreasing proportion of men (reducing male:female ratio) with increasing mean age of onset is statistically significant (*p* < 0.0001). The pseudo-*R*^2^ value for this association is 59.63%. The solid line is the fitted meta-regression back transformed from the logit scale. The dashed lines are the 95% confidence intervals for the mean association back transformed to the original scale.

For all phenotypes (53 reports of phenotypes from 24 studies), using a mixed effects meta-regression analysis, a statistically significant association between the sex ratio (proportion men) and mean age of onset was identified; the coefficient of age of onset in a model for logit (proportion men) was −0.0709, (95% CI −0.0900 to −0.0518) *p* < 0.0001, (Table 1). The pseudo-*R*^2^ value for this association was 59.63%. This indicates evidence of a linear decreasing male sex ratio with increasing age at symptom onset and that almost 60% of the variance in sex ratio is accounted for by age at onset. This effect is illustrated in the plot of the proportion of men versus mean age of onset in Figure 1.

**Table 1:** **Meta-regression analysis of relationship between mean age of onset and sex ratio in phenotypes of adult-onset dystonia**.

	Coefficient	SE	*Z* value	*p* Value	Confidence intervals	
**(A) META-REGRESSION ANALYSIS OF SEX RATIO (PROPORTION OF MEN) IN RELATION TO MEAN AGE OF ONSET OF ADULT-ONSET DYSTONIA**						
Intercept	2.8780	0.4487	6.4141	<0.0001	1.9986	3.7574
Age of onset	−0.0709	0.0098	−7.2655	<0.0001	−0.0900	−0.0518
**(B) META-REGRESSION ANALYSIS OF SEX RATIO (PROPORTION OF MEN) IN RELATION TO DYSTONIA PHENOTYPE WITH BLEPHAROSPASM AS THE REFERENCE**						
Intercept	−0.8387	0.1101	−7.6183	<0.0001	−1.0545	−0.6229
Cervical dystonia	0.351	0.1451	2.42	0.0155	0.067	0.6353
Focal hand dystonia	1.17	0.17	6.93	<0.0001	0.84	1.50
Laryngeal dystonia	0.047	0.19	0.243	0.808	−0.33	0.42
Musician’s dystonia	2.30	0.27	8.50	<0.0001	1.77	2.83
**(C) META-REGRESSION ANALYSIS OF SEX RATIO (PROPORTION OF MEN) IN RELATION TO BOTH MEAN AGE OF ONSET OF ADULT-ONSET DYSTONIA AND DYSTONIA PHENOTYPE WITH BLEPHAROSPASM AS THE REFERENCE**						
Intercept	4.7640	1.1084	4.2982	<0.0001	2.5916	6.9364
Age of onset	−0.1002	0.0197	−5.0840	<0.0001	0.1388	0.0616
Cervical dystonia	−1.0618	0.3013	−3.5239	0.0004	1.6523	0.4712
Focal hand dystonia	−0.4995	0.3556	−1.4046	0.1601	−1.1966	0.1975
Laryngeal dystonia	−0.8752	0.2386	−3.6685	0.0002	1.3429	0.4076
Musician’s dystonia	0.0306	0.5006	0.0611	0.9513	−0.9505	1.0117

*Meta-regression analysis of the two independent variables of interest: (a) mean age of onset of dystonia, (b) adult-onset dystonia phenotype, and (c) combined variables [(a) and (b)] with the sex ratio (proportion of men) as the dependent variable. Coefficients, SEs, p values, and 95% confidence intervals are shown; p values <0.05 are considered to be statistically significant*.

#### Meta-Regression Analysis of Relationship Between Sex Ratio and Phenotype at Presentation

Using the same dataset of 53 reports in a mixed effects meta-regression analysis, a statistically significant association between the sex ratio (proportion of men) and phenotype was identified; the coefficient of phenotypes in the model for logit (proportion men) are presented in Table 1. The pseudo-*R*^2^ value for this association was 81%.

#### Meta-Regression Analysis of Relationship Between Sex Ratio and Both Mean Age of Onset and Phenotype at Presentation

Using the same dataset of 53 reports in a mixed effect meta-regression analysis, a statistically significant association between the sex ratio (proportion of men) and both age at onset and phenotype was identified (Table 1). The pseudo-*R*^2^ value for this association was 92.83%. This indicates that the almost 93% of the variance in sex ratio (proportion men) in adult-onset dystonia is accounted for by the combination of mean age at onset and phenotype. A model, which included interactions between phenotype and age, was also fitted but these were not found to be statistically significant.

### Study B: Age- and Sex-Related Effects on Temporal Discrimination in Unaffected Relatives

**Figure 2:**
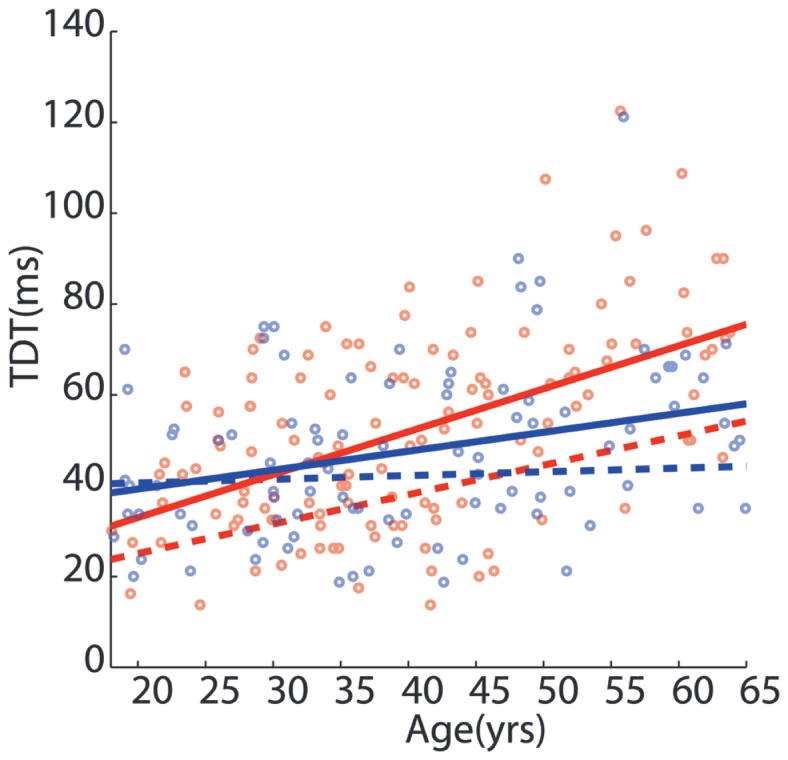
**Sexual dimorphism in temporal discrimination in unaffected first-degree relatives of cervical dystonia patients**. The effect of age and gender on the temporal discrimination threshold (in milliseconds) in 220 unaffected first-degree relatives of patients with cervical dystonia. Each open circle represents an individual relative’s temporal discrimination threshold (men: blue; women: red). The solid lines represent the regression fit of the data for men (blue) and women (red) of temporal discrimination thresholds with age. The broken red and blue lines indicate the regression fit of the temporal discrimination threshold for 175 healthy control participants [from Ref. (15)]. The sexually dimorphic effects of age on the temporal discrimination thresholds in men and women are statistically significant for both the unaffected first-degree relatives and the healthy control participants.

The linear regression fits of TDT as a function of age for women (red) and men (blue) in 220 unaffected first-degree relatives (solid lines) and 175 control participants (broken lines) are illustrated in Figure 2. The regression for female relatives showed that age explained a significant amount of the variance in the TDT values [*F*(1,124) = 45.758, *p* < 0.001, *R*^2^ = 0.271], with a significant intercept 12.7 ms, {*t*(124) = 2.08, *p* < 0.05; 95% CI [8.254, 24.814]}, and a significant β = 0.985 ms, {*t*(124) = 6.76, *p* < 0.001; 95% CI [0.697, 1.273]} (Table S2 in Supplementary Material). In male relatives, there was a significant relationship between age and TDT values [*F*(1,94) = 8.9, *p* < 0.005, *R*^2^ = 0.087] demonstrating a significant intercept 31.09 ms, {*t*(94) = 5.065, *p* < 0.001; 95% CI [18.9, 43.283]}, and a significant β = 0.437 ms, {*t*(94) = 2.984, *p* < 0.005; 95% CI [0.22, 0.738]}. Comparison of regression analysis in relatives between women and men revealed a significantly lower intercept in women than men −18.382 ms {*t*(218) = −2.11, *p* < 0.05; 95% CI [−35.49, −1.273]} and significant β value for the interaction of sex and age 0.548 ms {*t*(218) = 2.646, *p* < 0.01; 95% CI [0.14, 0.956]}, with a 1 ms increase in the TDT every year in women; in men an increase by 0.5 ms each year. The regression line for men and the regression line for women intersected at approximately 31 years of age. As with the unaffected relatives, the regression analysis between men and women in the 175 control participants showed an effect of sex and an interaction of sex and age [see Table S2 in Supplementary Material and Ref. (15)]. Comparison of regression fits of control participants and relatives resulted in a significant difference in the β value for the interaction of age and group −0.343 {*t*(392) = −2.293, *p* < 0.05; 95% CI [−0.594, −0.049]}, with no significant effect of group (Table S2 in Supplementary Material). While both unaffected relatives and control participants at age 20 years have similar TDTs, with increasing age the TDT worsens by 0.34 ms/year in relatives compared to healthy control participants.

### Study C: Cervical Dystonia: Differential Mean Age of Onset by Sex

**Figure 3:**
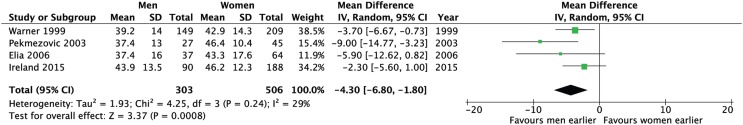
**Meta-analysis of mean age of onset of cervical dystonia in men and women: meta-analysis of the mean age of onset by sex in our cohort of 278 cervical dystonia patients and 3 other studies which reported mean age of onset of cervical dystonia for both sexes (**16–18). This analysis demonstrated a significantly earlier mean age of onset for men than women. The mean age of onset of cervical dystonia was significantly earlier by 4.30 years (95% CI; 1.80–6.80) in men than women (*p* = 0.0008).

Meta-analysis of the mean age of onset by sex in our cervical dystonia cohort in 278 patients (188 women) and 3 other studies (18–20), which reported mean age of onset of cervical dystonia for both sexes is shown in Figure 3. In each of the four cohorts (total: 303 men and 506 women), men had an earlier mean age of onset than women; in the meta-analysis, the mean age of onset of cervical dystonia was significantly earlier by 4.3 years (95% CI; 1.8–6.8) in men than women (*p* = 0.0008). Thus, despite the overall preponderance of women in cervical dystonia, men have a significantly earlier mean age of onset than women.

## Discussion

### Age-Related Sexual Dimorphism in Disease Penetrance in AOIFD

The male:female sex ratio (proportion of men) in AOIFD decreases with increasing mean age at onset; this association is highly significant and mean age of onset accounts for almost 60% of the variance in the proportion of men. AOIFD with onset below the age of 40 years affects predominantly men in focal hand dystonia and musician’s dystonia. After 40–45 years of age, the AOIFD phenotypes predominantly affect women and, importantly, with increasing age there is a steady linear decrease in the proportion of men affected (Figure 1). The cervical dystonia phenotype is of particular interest because, although overall it affects women 1.5–2.0 times more frequently than men, men have a mean age of onset approximately 4 years earlier than women (Figure 3).

### Age-Related Sexual Dimorphism in Temporal Discrimination in Unaffected Relatives

In both unaffected first-degree relatives and healthy control participants (15), temporal discrimination shows a significant effect of sex, age, and an interaction of sex and age. Female relatives and female control participants under the age of 40 years have faster temporal discrimination than age-matched male relatives and controls. Overall temporal discrimination worsens as a function of age but this is more pronounced in women than men; the TDT in women deteriorates two times faster than in men. Sexual dimorphism of temporal discrimination is thus seen both in unaffected relatives (Figure 2) and in healthy participants (15). However, the effect of age on the TDT of unaffected relatives was more marked than in healthy participants, worsening by 0.34 ms/year more in relatives than controls. We postulate that this increased worsening with age in unaffected relatives indicates an effect of non-manifesting gene carriage on the TDT endophenotype; approximately half of these unaffected first-degree relatives are non-manifesting carriers of susceptibility genes for adult-onset dystonia (13, 14).

### Age-Related Sexual Dimorphism in Ambient GABA

GABA levels, measured by magnetic resonance spectroscopy (MRS), in the frontal lobes in healthy participants show age-related sexual dimorphism (21). Under 40 years of age women have higher GABA levels than men. However, with increasing age, women show more marked progressive reduction in frontal lobe GABA than men, so that after 40 years of age men have increasingly higher frontal lobe GABA than women.

Deficient inhibitory GABAergic mechanisms are considered to be important in the pathogenesis of dystonia. Cortical and subcortical GABA levels are reduced contralateral to the affected hand in patients with focal hand dystonia (22). Alterations in inhibitory circuits in dystonia have been noted at spinal cord, brainstem, and cortical levels (23). Transcranial magnetic stimulation studies have shown impaired intra-cortical inhibition in both hemispheres in focal hand dystonia, even though symptoms were present on only one side of the body (24). Deficient inhibition may be responsible for excessive and overflow movements seen in AOIFD (24, 25).

### Age, Cognition, and GABA

Normal aging is associated with a decline in aspects of cognitive function, including slowing in performance of visual orientation discrimination (26), visual face matching (27), three-dimensional shape determination (28), and motion direction detection (29). Even in young healthy individuals, variation in cortical GABA levels has been found to relate to a number of psychophysical measures, including tactile discrimination thresholds (30) and luminance grating orientation (31), with poorer performance associated with regionally reduced GABA levels. The ability to respond to a visual target speedily in the presence of a visual distraction has been linked to frontal lobe GABA measured by MRS (32). Reduced GABAergic inhibition, either age-related or due to physiological variation, results in blunting of stimulus–response tuning specificity and task performance. In animals, degradation in these visual discriminatory abilities, associated with a lower signal to noise ratio in the striate cortex, has been linked to a reduction (by 45–60%) of GABA immuno-reactive neurones in the older animal (33, 34). Age-related reduction in GABA has been noted in the inferior colliculus (35), and the hippocampus in rats (36).

### Linking Clinical and Laboratory Observations

We postulate that the age-related sexual dimorphism in (a) disease penetrance in AOIFD, (b) temporal discrimination, and (c) ambient cerebral GABA are linked and illuminate pathogenic mechanisms in AOIFD. The underlying hypothesis is that the efficiency of temporal discrimination is a measure of effective temporal tuning in the basal ganglia/superior colliculus, and relates to GABAergic inhibitory mechanisms. Experimental work in animals indicates that effective GABAA and GABAB inhibitory mechanisms are necessary for the sharp onset and offset of responses to visual stimuli in the superficial laminae of the superior colliculus (37–39). While GABA may be measured in large cortical structures by MRS, there is no method, as yet, to measure ambient or synaptic GABA in a structure as small as the superior colliculus in man *in vivo*. Thus, the hypothesis that faster temporal discrimination reflects more effective GABAergic inhibition in man remains likely, but unproven. The clinical relevance to adult-onset focal dystonia is that age-related sexual dimorphism in ambient GABA (causing the observed sexual dimorphism in temporal discrimination), results in the age-related sexual dimorphism in AOIFD disease penetrance.

**Figure 4:**
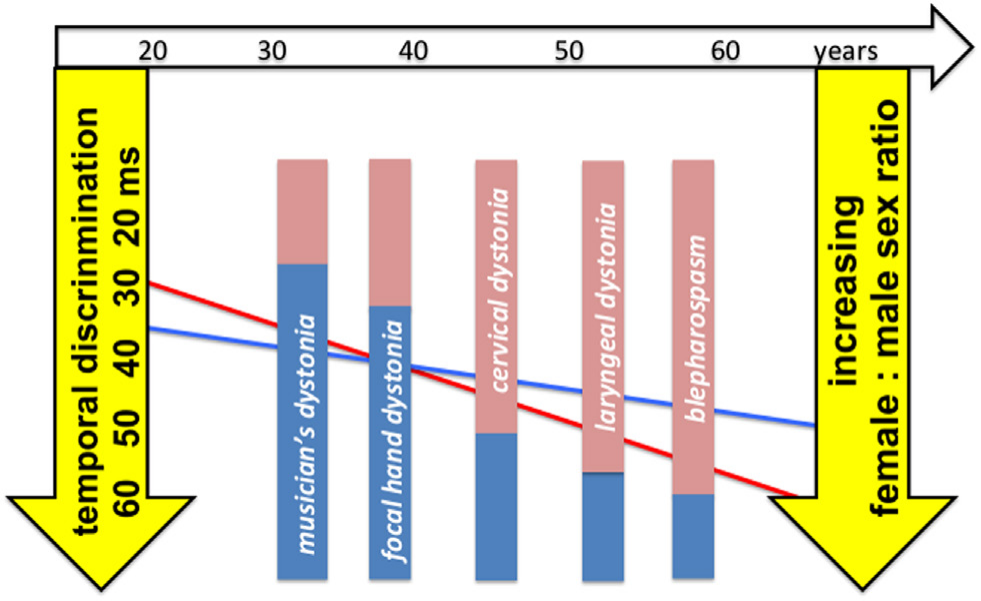
**Graphic illustrating the relationship between age-related sexual dimorphism in temporal discrimination thresholds, mean age of onset and sex ratios in phenotypes of adult-onset isolated focal dystonia**. Temporal discrimination (in milliseconds) is inverted on the left *Y*-axis so that worse discrimination is lower on the axis. The *X*-axis represents age in years (open arrow at the top of the figure). Each of the five phenotypes (musician’s dystonia, focal hand dystonia, cervical dystonia, laryngeal dystonia, and blepharospasm) are represented by filled bars, positioned at their mean age of onset, with the sex ratio for each phenotype illustrated by the colored inserts (pink for women: blue for men) (right *Y*-axis); the increasing female:male sex ratio with age is illustrated by the relative size of the filled bars. The regression lines (red for women; blue for men) from the analysis of the temporal discrimination thresholds (TDTs) in the unaffected first-degree relatives are drawn to illustrate the age-related sexual dimorphism of the TDT in relationship to the increasing female predominance with age of the phenotypes.

Arising from these observations, we hypothesize that, in a person with a genetic susceptibility to AOIFD, penetrance varies according to gender and age (Figure 4): (i) under 40 years of age, women have faster temporal discrimination than men, have more ambient GABA and are resistant to disease penetrance; thus, the observed male predominance in focal hand dystonia and musician’s dystonia. (ii) The increased age-related rate of deterioration in temporal discrimination in women reflects faster reducing ambient GABA levels in women than men. After 40–45 years, women have increasingly worse temporal discrimination and increasingly lower ambient GABA than men. Older women are thus more susceptible to disease penetrance and thus the observed progressively increasing age-related female predominance in adult-onset dystonia phenotypes presenting after the age of 40 years (Figure 4).

## Author Contributions

Conception (MH); design (MH, RR, and SO); organization (JB, LW, IB, and EM); statistical analysis (CW, JB, and LW); execution (IB, LW, EM, JB, DH, TL, HM, FM, and RW); review and critique (RR, MH, and JB); manuscript preparation: writing the first draft (MH); review and critique (all authors).

## Conflict of Interest Statement

John S. Butler, Ines M. Beiser, Laura Williams, Eavan McGovern, Dan G. Healy, Helena Moore, Richard Walsh, and Cathal Walsh have no disclosures. Michael Hutchinson: serves as associate editor of the Multiple Sclerosis Journal, has received speaker’s honoraria from Biogen Idec, Bayer-Schering, and Novartis and receives research grants from Dystonia Ireland, the Health Research Board of Ireland (CSA-2012-5), Foundation for Dystonia Research (Belgium), and the Irish Institute of Clinical Neuroscience. Tim Lynch reports receiving educational grants from Bayer Schering, Biogen Idec, Lundbeck, Medtronic; research grants from Irish Institute of Clinical Neuroscience, Mater College, PRTL1 funding. Speaker’s honoraria from Novartis. UCB Pharma, Teva, Merck Serono, and Biogen Idec. Sean O’Riordan reports receiving a speaker’s honararium from Abbvie. Richard B. Reilly is in receipt of research grants from Health Research Board Ireland: FP7-288914-VERVE, “VERVE: Vanquishing fear and apathy through E-inclusion: personalized and populated Realistic Virtual Environments for clinical, home, and mobile platforms” co-PI with Prof. C. O’Sullivan and Prof. F. Newell. Total project €4.6M Oct 2012–Oct 2014; HRB: “INCA: Inhaler device for objective analysis of medication adherence” with Prof. R. Costello, Beaumont Hospital. €120,987, Oct 2013–Oct 2016. €132,894 from Cochlear Ltd. for objective analysis of temporal and spectral processing in Cochlear Implant users. Fiona Molloy has received speaker’s honorarium from Allergan and Ipse.

## Abbreviations

AOIFD, adult-onset isolated focal dystonia; TDT, temporal discrimination threshold.

## References

[1] Soland VL Bhatia KP Marsden CD. Sex prevalence of focal dystonias. J Neurol Neurosurg Psychiatry (1996) 60:204–5. 10.1136/jnnp.60.2.204 8708656 PMC1073807

[2] Albanese A Bhatia K Bressman SB Delong MR Fahn S Fung VS Phenomenology and classification of dystonia: a consensus update. Mov Disord (2013) 28:863–73. 10.1002/mds.25475 23649720 PMC3729880

[3] Waddy HM Fletcher NA Harding AE Marsden CD. A genetic study of idiopathic focal dystonias. Ann Neurol (1991) 29:320–4. 10.1002/ana.410290315 2042948

[4] Leube B Kessler KR Goecke T Auburger G Benecke R. Frequency of familial inheritance among 488 index patients with idiopathic focal dystonia and clinical variability in a large family. Mov Disord (1997) 12:1000–6. 10.1002/mds.870120625 9399227

[5] Defazio G Berardelli A Hallett M. Do primary adult-onset focal dystonias share aetiological factors? Brain (2007) 130:1183–93. 10.1093/brain/awl355 17242025

[6] Brancati F Defazio G Caputo V Valente EM Pizzuti A Livrea P Novel Italian family supports clinical and genetic heterogeneity of primary adult-onset torsion dystonia. Mov Disord (2002) 17:392–7. 10.1002/mds.10077 11921130

[7] O’Riordan S Raymond D Lynch T Saunders-Pullman R Bressman SB Daly L Age at onset as a factor in determining the phenotype of primary torsion dystonia. Neurology (2004) 63:1423–6. 10.1212/01.WNL.0000142035.26034.C2 15505159

[8] Bradley D Whelan R Walsh R Reilly RB Hutchinson S Molloy F Temporal discrimination threshold: VBM evidence for an endophenotype in adult-onset primary torsion dystonia. Brain (2009) 132:2327–35. 10.1093/brain/awp156 19525326

[9] Bradley D Whelan R Walsh R O’Dwyer J Reilly R Hutchinson S Comparing endophenotypes in adult-onset primary torsion dystonia. Mov Disord (2010) 25:84–90. 10.1002/mds.22889 19938165

[10] Bradley D Whelan R Kimmich O O’Riordan S Mulrooney N Brady P Temporal discrimination thresholds in adult-onset primary torsion dystonia: an analysis by task type and by dystonia phenotype. J Neurol (2012) 259:77–82. 10.1007/s00415-011-6125-7 21656045

[11] Kimmich O Bradley D Whelan R Mulrooney N Reilly RB Hutchinson S Sporadic adult onset primary torsion dystonia is a genetic disorder by the temporal discrimination test. Brain (2011) 134:2656–63. 10.1093/brain/awr194 21840890

[12] Kimmich O Molloy A Whelan R Williams L Bradley D Balsters J Temporal discrimination, a cervical dystonia endophenotype: penetrance and functional correlates. Mov Disord (2014) 29:804–11. 10.1002/mds.25822 24482092

[13] Hutchinson M Kimmich O Molloy A Whelan R Molloy F Lynch T The endophenotype and the phenotype: temporal discrimination and adult onset dystonia. Mov Disord (2013) 28:1766–74. 10.1002/mds.25676 24108447

[14] Hutchinson M Isa T Molloy A Kimmich O Williams L Molloy F Cervical dystonia: a disorder of the midbrain network for covert attentional orienting. Front Neurol (2014) 5:54. 10.3389/fneur.2014.00054 24803911 PMC4009446

[15] Williams LJ Butler JS Molloy A McGovern E Beiser I Kimmich O Young women do it better: sexual dimorphism in temporal discrimination. Front Neurol (2015) 6:160. 10.3389/fneur.2015.00160 26217303 PMC4497309

[16] Viechtbauer W Conducting meta-analyses in R with the metafor package. J Stat Softw (2010) 36:1–48. 10.18637/jss.v036.i03

[17] Raudenbush SW Analyzing effect sizes: random effects models. 2nd ed In: Cooper H Hedges LV Valentine JC, editors. The Handbook of Research Synthesis and Meta-Analysis. New York, NY: Russell Sage Foundation (2009). 295 p.

[18] Warner T Ben-Shlomo Y Group ESoDiEEC. Sex-related influences on the frequency and age of onset of primary dystonia. Epidemiologic Study of Dystonia in Europe (ESDE) Collaborative Group. Neurology (1999) 53:1871–3. 10.1212/WNL.53.8.1871 10563645

[19] Pekmezović T Ivanović N Svetel M Nalić D Smiljković T Raicević R Prevalence of primary late-onset focal dystonia in the Belgrade population. Mov Disord (2003) 18:1389–92. 10.1002/mds.10615 14639690

[20] Elia AE Filippini G Bentivoglio AR Fasano A Ialongo T Albanese A. Onset and progression of primary torsion dystonia in sporadic and familial cases. Eur J Neurol (2006) 13:1083–8. 10.1111/j.1468-1331.2006.01387.x 16987160

[21] Gao F Edden RA Li M Puts NA Wang G Liu C Edited magnetic resonance spectroscopy detects an age-related decline in brain GABA levels. Neuroimage (2013) 78:75–82. 10.1016/j.neuroimage.2013.04.012 23587685 PMC3716005

[22] Levy LM Hallett M. Impaired brain GABA in focal dystonia. Ann Neurol (2002) 51:93–101. 10.1002/ana.10073 11782988

[23] Berardelli A Rothwell JC Hallett M Thompson PD Manfredi M Marsden CD. The pathophysiology of primary dystonia. Brain (1998) 121:1195–212. 10.1093/brain/121.7.1195 9679773

[24] Bütefisch CM Boroojerdi B Chen R Battaglia F Hallett M. Task-dependent intracortical inhibition is impaired in focal hand dystonia. Mov Disord (2005) 20:545–51. 10.1002/mds.20367 15641012 PMC1457024

[25] Hallett M. Neurophysiology of dystonia: the role of inhibition. Neurobiol Dis (2011) 42:177–84. 10.1016/j.nbd.2010.08.025 20817092 PMC3016461

[26] Betts LR Sekuler AB Bennett PJ. The effects of aging on orientation discrimination. Vision Res (2007) 47:1769–80. 10.1016/j.visres.2007.02.016 17466355

[27] Wilson HR Mei M Habak C Wilkinson F. Visual bandwidths for face orientation increase during healthy aging. Vision Res (2011) 51:160–4. 10.1016/j.visres.2010.10.026 21074549

[28] Norman JF Norman HF Pattison K Taylor MJ Goforth KE. Aging and the depth of binocular rivalry suppression. Psychol Aging (2007) 22:625–31. 10.1037/0882-7974.22.3.625 17874959

[29] Betts LR Taylor CP Sekuler AB Bennett PJ Aging reduces center surround antagonism in visual motion processing. Neuron (2005) 45:361–6. 10.1016/j.neuron.2004.12.041 15694323

[30] Puts NA Edden RA Evans CJ McGlone F McGonigle DJ. Regionally specific human GABA concentration correlates with tactile discrimination thresholds. J Neurosci (2011) 31:16556–60. 10.1523/JNEUROSCI.4489-11.2011 22090482 PMC3374929

[31] Edden RAE Muthukumaraswamy SD Freeman TCA Singh KD. Orientation discrimination performance is predicted by GABA concentration and gamma oscillation frequency in human primary visual cortex. J Neurosci (2009) 29:15721–6. 10.1523/JNEUROSCI.4426-09.2009 20016087 PMC6666191

[32] Sumner P Edden RA Bompas A Evans CJ Singh KD. More GABA, less distraction: a neurochemical predictor of motor decision speed. Nat Neurosci (2010) 13:825–7. 10.1038/nn.2559 20512136

[33] Hua T Li X He L Zhou Y Wang Y Leventhal AG. Functional degradation of visual cortical cells in old cats. Neurobiol Aging (2006) 27:155–62. 10.1016/j.neurobiolaging.2004.11.012 16298251

[34] Hua T Kao C Sun Q Li X Zhou Y. Decreased proportion of GABA neurons accompanies age-related degradation of neuronal function in cat striate cortex. Brain Res Bull (2008) 75:119–25. 10.1016/j.brainresbull.2007.08.001 18158105

[35] Ouda L Syka J. Immunocytochemical profiles of inferior colliculus neurons in the rat and their changes with aging. Front Neural Circuits (2012) 6:68. 10.3389/fncir.2012.00068 23049499 PMC3448074

[36] Stanley EM Fadel JR Mott DD. Interneuron loss reduces dendritic inhibition and GABA release in hippocampus of aged rats. Neurobiol Aging (2012) 33:e1–13. 10.1016/j.neurobiolaging.2010.12.014 21277654 PMC3110542

[37] Kaneda K Phongphanphanee P Katoh T Isa K Yanagawa Y Obata K Regulation of burst activity through pre- and postsynaptic GABAB receptors in mouse superior colliculus. J Neurosci (2008) 28:816–27. 10.1523/JNEUROSCI.4666-07.2008 18216190 PMC6671012

[38] Kaneda K Isa K Yanagawa Y Isa T Nigral inhibition of GABAergic neurons in mouse superior colliculus. J Neurosci (2008) 28:11071–8. 10.1523/JNEUROSCI.3263-08.2008 18945914 PMC6671385

[39] Kaneda K Isa T. GABAergic mechanisms for shaping transient visual responses in the mouse superior colliculus. Neuroscience (2013) 235:129–40. 10.1016/j.neuroscience.2012.12.061 23337535

