# Supplementary material for: Age-Related Sexual Dimorphism in Temporal Discrimination and in Adult-Onset Dystonia Suggests GABAergic Mechanisms
Source: Front Neurol. 2015 Dec 14;6:258. doi: 10.3389/fneur.2015.00258 (PMC4677337; doi:10.3389/fneur.2015.00258)
Supplement: Supplementary file 1 [file Table_1.DOCX]

**Supplementary Table 1.**

**Supplementary Table 1: Table of mean age onset and sex ratio in adult onset dystonia:** 53 reports of mean age of onset, female: male sex ratio and

proportion of men in five phenotypes (blepharospasm, cervical dystonia, focal hand dystonia, musician’s dystonia and laryngeal dystonia) of adult onset isolated focal dystonia. These 53 cohorts are from 24 published studies (references below listed) by first author in column 1 and city or country of origin in column 2.

| **study (first author, year)** | **origin** | **number** | **mean age onset**  **in years (SD)** | **female:**  **male ratio** | **proportion men** |
| --- | --- | --- | --- | --- | --- |
| **blepharospasm** | | | | | |
| Acquino, 2012 | Brazil | 65 | 54.1 (11) | 2.8 | 0.26 |
| Asgeirsson, 2006 | Iceland | 9 | 54.8 (14.3) | 2 | 0.33 |
| Butler, 2004 | North England | 219 | 54.7 (NA) | 2.8 | 0.26 |
| Castelon K, 2002 | Germany | 41 | 57.1 (10.9) | 2.2 | 0.31 |
| Cossu, 2006 | Italy | 53 | 58.5 (13) | 2.53 | 0.28 |
| Dhaenens, 2005 | Lille, France | 48 | 51.3 (8.3) | 1.38 | 0.42 |
| Elia, 2006 | Italy | 230 | 56.4 (11.9) | 2.83 | 0.26 |
| Groen, 2012 | Netherlands | 97 | 54.0 (12.1) | 1.75 | 0.36 |
| Le, 2003 | Norway | 24 | 56.8 (NA) | 5 | 0.17 |
| Leube, 1997 | Germany | 63 | 56 (NA) | 2.5 | 0.29 |
| Marsden, 1976 | UK | 13 | 57 (NA) | 1.6 | 0.38 |
| Martino, 2012 | Italy | 280 | 57 (10) | 2.84 | 0.26 |
| Pekmezovic, 2003 | Serbia | 23 | 57.9 (11.0) | 2.3 | 0.3 |
| Warner, 1999 | Europe | 210 | 58.8 | 2.3 | 0.3 |
| **cervical dystonia** | | | | | |
| Asgeirsson, 2006 | Iceland | 33 | 41.7 (14.4) | 2.3 | 0.3 |
| Butler, 2004 | England | 566 | 42.1 (NA) | 2.1 | 0.32 |
| Castelon K, 2002 | Germany | 72 | 41.6 (14.1) | 1.3 | 0.43 |
| Chan, 1991 | USA | 266 | 41.3 NA) | 1.9 | 0.34 |
| Cheng, 1996 | USA | 122 | 44.3 (14.7) | 2.3 | 0.3 |
| Dhaenens, 2005 | Lille, France | 47 | 38.6 (12.8) | 1.79 | 0.36 |
| Elia, 2006 | Italy | 101 | 41.1 (17.1) | 1.73 | 0.37 |
| Groen, 2012 | Netherlands | 432 | 41.9 (14.1) | 1.34 | 0.43 |
| Le, 2003 | Norway | 66 | 41.2 (NA) | 1.9 | 0.34 |
| Leube, 1997 | Germany | 396 | 41 (12) | 1.39 | 0.42 |
| Maniak, 2003 | Germany | 89 | 41 (13.5) | 1.47 | 0.4 |
| Martino, 2012 | Italy | 111 | 44 (15) | 2.7 | 0.27 |
| Nutt, 1988 | USA | 11 | 45 (NA) | 4.5 | 0.18 |
| Pekmezovic, 2003 | Serbia | 72 | 43.3 (12.1) | 1.7 | 0.37 |
| Warner, 1999 | Europe | 358 | 41.4 (NA) | 1.4 | 0.42 |
| **Focal hand dystonia** | | | | | |
| Abbruzzese, 2008 | Italy | 26 | 39.1 (12.6) | 0.86 | 0.54 |
| Asgeirsson, 2006 | Iceland | 23 | 33.5 (11.3) | 0.53 | 0.64 |
| Butler, 2004 | North England | 93 | 37.1 (NA) | 0.71 | 0.58 |
| Elia, 2006 | Italy | 71 | 36.4 (17.3) | 0.45 | 0.69 |
| Groen, 2012 | Netherlands | 68 | 39.2 (10.8) | 0.55 | 0.65 |
| Jedynak, 2001 | Belgium | 65 | 36.3 (NA) | 0.55 | 0.65 |
| Le, 2003 | Oslo | 12 | 50.3 (NA) | 2.0 | 0.33 |
| Leube, 1997 | Germany | 27 | 37 (9) | 0.5 | 0.67 |
| Maniak, 2003 | North Germany | 16 | 43.5 (12) | 0.6 | 0.63 |
| Pekmezovic, 2003 | Serbia | 8 | 39.8 (11.7) | 0.6 | 0.63 |
| Roze, 2009 | Paris | 104 | 44 (NA) | 1.6 | 0.38 |
| Warner, 1999 | Europe | 94 | 41.4 (NA) | 0.77 | 0.56 |
| **musician’s dystonia** | | | | | |
| Conti, 2008 | New York | 61 | 37.1 (12.3) | 0.22 | 0.82 |
| Newmark, 1987 | Boston | 59 | 34 (NA) | 0.4 | 0.71 |
| Rosset-Llobet, 2015 | Barcelona | 101 | 29.3 (10.8) | 0.13 | 0.88 |
| **laryngeal dystonia** | | | | | |
| Asgeirsson, 2006 | Iceland | 17 | 50.1 (14.7) | 2.4 | 0.29 |
| Butler, 2004 | North England | 129 | 47.4 (NA) | 4.6 | 0.18 |
| Castelon K, 2002 | Munich | 13 | 48.0 (11.2) | 1.3 | 0.43 |
| Cheng, 1996 | California | 67 | 42.3 (17.9) | 1.91 | 0.34 |
| Elia, 2006 | Italy | 38 | 47.7 (21.1) | 2.45 | 0.29 |
| Groen, 2012 | Netherlands | 79 | 46.4 (15.2) | 1.67 | 0.37 |
| Le, 2003 | Oslo | 14 | 49.6 (NA) | 1.3 | 0.43 |
| Pekmezovic, 2003 | Serbia | 13 | 46.3 (18.7) | 1.6 | 0.38 |
| Warner, 1999 | Europe | 40 | 50.7 (NA) | 2.6 | 0.28 |

***Studies referenced in supplementary table 1 and included in the meta-regression analysis of mean age of onset and sex ratio (study A).***

Abbruzzese G, Berardelli A, Girlanda P, Marchese R, Martino D, Morgante F, et al. Long-term assessment of the risk of spread in primary late-onset focal dystonia. J Neurol Neurosurg Psychiatry 2008;79, 392-6.

Aquino CC, Felício AC, Castro PC, Oliveira RA, Silva SM, Borges V, et al. Clinical features and treatment with botulinum toxin in blepharospasm: a 17-year experience. Arq Neuropsiquiatr 2012;70:662-6.

Asgeirsson H, Jakobsson F, Hjaltason H, Jonsdottir H, Svein-Bjornsdottir S. 2006. Prevalence study of primary dystonia in Iceland. Mov. Disord 2006; 21: 293–8.

Butler AG, Duffey PO, Hawthorne MR, Barnes MP. An epidemiologic survey of dystonia within the entire population of northeast England over the past nine years. Adv Neurol 2004; 94: 9509.

Castelon Konkiewitz E, Trender-Gerhard I, Kamm C, Warner T, Ben-Shlomo Y, Gasser T, Conrad B, Ceballos-Baumann AO. 2002. Service-based survey of dystonia in Munich. Neuroepidemiology 2002;21: 202-6.

Chan J, Brin, MF, Fahn S. Idiopathic cervical dystonia: clinical characteristics. Mov Disord 1991;6:119-26.

Cheng JT, Liu A, Wasmuth J, Liu BP, Truong D. Clinical evidence of genetic anticipation in adult-onset idiopathic dystonia. Neurology 1996;47:215-9.

Conti AM, Pullman S, Frucht SJ. The hand that has forgotten its cunning--lessons from musicians' hand dystonia. Mov Disord 2008; 23:1398-406.

Cossu G, Mereu A, Deriu M, Melis M, Molari A, Melis G, et al. 2006. Prevalence of primary blepharospasm in Sardinia, Italy: a service-based survey. Mov Disord 2006; 21:2005-8.

Dhaenens CM, Krystkowiak P, Douay X, Charpentier P, Bele S, Destée A,

et al. Clinical and genetic evaluation in a French population presenting with primary focal dystonia. Mov Disord 2005;20:822-5.

Elia AE, Filippini G, Bentivoglio AR, Fasano A, Ialongo T, Albanese A. Onset and progression of primary torsion dystonia in sporadic and familial cases. Eur. J. Neurol 2006;13:1083-8.

Groen JL, Kallen MC, van de Warrenburg BP, Speelman JD, van Hilten, J.J., Aramideh, M. et al. Phenotypes and genetic architecture of focal primary torsion dystonia. J Neurol Neurosurg Psychiatry 2012; 83:1006-11.

Jedynak PC, Tranchant C, de Beyl DZ. Prospective clinical study of writer's cramp. Mov Disord 2001;16:494-9.

Le KD, Nilsen B, Dietrichs E. Prevalence of primary focal and segmental dystonia in Oslo. Neurology 2003; 61:1294-6.

Leube B, Kessler KR, Goecke T, Auburger G, Benecke R. Frequency of familial inheritance among 488 index patients with idiopathic focal dystonia and clinical variability in a large family. Mov Disord 1997;12:1000-6.

Maniak S, Sieberer M, Hagenah J, Klein C, Vieregge P. Focal and segmental primary dystonia in north-western Germany--a clinico-genetic study. Acta Neurol Scand 2003; 107: 228-32.

Marsden CD. Blepharospasm-oromandibular dystonia syndrome (Brueghel's syndrome). A variant of adult-onset torsion dystonia? J Neurol Neurosurg Psychiatry 1976;39:1204-9.

Martino D, Berardelli A, Abbruzzese G, Bentivoglio AR, Esposito M, Fabbrini G, et al. Age at onset and symptom spread in primary adult-onset blepharospasm and cervical dystonia. Mov Disord 2012; 227:1447-50.

Newmark J, Hochberg FH. Isolated painless manual incoordination in 57 musicians. J. Neurol Neurosurg Psychiatry 1987; 50:291-5.

Nutt JG, Muenter MD, Aronson A, Kurland LT, Melton LJ. Epidemiology of focal and generalized dystonia in Rochester, Minnesota. Mov Disord 1988;3:188-94.

Pekmezovic T, Ivanovic N, Svetel M, Nalic D, Smiljkovic T, Raicević R, et al. Prevalence of primary late-onset focal dystonia in the Belgrade population. Mov Disord 2003;18:1389-92

Rosset-Llobet J, Candia V, Fàbregas S, Ray W, Pascual-Leone A. Secondary motor disturbances in 101 patients with musician's dystonia. J. Neurol Neurosurg. Psychiatry 2007;78: 949–53.

Roze E, Soumare A, Pironneau I, Sangla S, de Cock VC, Astorquiza,

A, et al. Case-control study of writer's cramp. Brain 2009;132:756-64.

Warner T, Ben-Shlomo Y, Group ESoDiEEC. Sex-related influences on the frequency and age of onset of primary dystonia. Epidemiologic Study of Dystonia in Europe (ESDE) Collaborative Group. Neurology 1999;53:1871-3.
