# Supplementary material for: Age-Related Sexual Dimorphism in Temporal Discrimination and in Adult-Onset Dystonia Suggests GABAergic Mechanisms
Source: Front Neurol. 2015 Dec 14;6:258. doi: 10.3389/fneur.2015.00258 (PMC4677337; doi:10.3389/fneur.2015.00258)
Supplement: Supplementary file 2 [file Table_2.DOCX]

**Supplementary Table 2: Regression analysis of age and sex on temporal discrimination thresholds.** Regression analysis of the two independent variables of interest, age and sex, on temporal discrimination thresholds in: (a) 220 unaffected first-degree relatives (125 women, 95 men) of patients with cervical dystonia; (b) 175 healthy control participants (88 women, 87 men) and (c) the independent variable, age, on temporal discrimination thresholds between groups (a) and (b). Coefficients, standard errors (SEs), p values and 95% confidence intervals are shown; p values <0.05 are considered to be statistically significant.

**Supplementary Table 2: Result of regression analysis of age and sex on temporal discrimination thresholds.**

| ***a) regression analysis of temporal discrimination thresholds***  ***in 220 unaffected relatives*** | | | | | | |
| --- | --- | --- | --- | --- | --- | --- |
| ***men*** *R^2^=0.087, F(1,94)=8.9, p<0.005* | | | | | | |
|  | coefficient | SE | t value | p value | 95% confidence intervals | |
| Intercept | 31.09 | 6.139 | 5.065 | < 0.001 | 18.902 | 43.283 |
| Age | 0.437 | 0.147 | 2.984 | < 0.005 | 0.22 | 0.738 |
| ***women*** *R^2^=0.271, F(1,124)=45.758, p<0.001* | | | | | | |
| intercept | 12.7 | 6.112 | 2.08 | <0.05 | 8.254 | 24.814 |
| age | 0.985 | 0.146 | 6.76 | < 0.001 | 0.697 | 1.273 |
| ***interaction of age and sex*** *R^2^=0.21, F(3,219)=19.17, p<0.001* | | | | | | |
| Intercept | 31.09 | 6.244 | 4.996 | <0.001 | 18.827 | 43.361 |
| Age | 0.437 | 0.149 | 2.943 | <0.005 | 0.144 | 0.730 |
| Sex | -18.382 | 8.68 | -2.11 | <0.05 | -35.491 | -1.273 |
| Sex*Age | 0.548 | 0.207 | 2.646 | <0.01 | 0.14 | 0.956 |
| ***b) regression analysis of temporal discrimination thresholds***  ***in 175 healthy participants*** | | | | | | |
| ***men*** *R^2^=0.087, F(1,86)=0.24, p=0.56* | | | | | | |
|  | coefficient | SE | t value | p value | 95% confidence intervals | |
| intercept | 38.97 | 5.939 | 6.561 | < 0.001 | 27.160 | 50.776 |
| Age | 0.082 | 0.140 | 0.583 | 0.562 | -0.197 | 0.36 |
| ***women*** *R^2^=0.197, F(1,87)=21.159, p<0.001* | | | | | | |
| intercept | 12.7 | 6.112 | 2.08 | <0.05 | -8.254 | 24.814 |
| age | 0.985 | 0.146 | 6.76 | < 0.0001 | 0.697 | 1.273 |
| ***interaction of age and sex*** *R^2^=0.127, F(3,174)=8.305, p<0.001* | | | | | | |
| intercept | 38.96 | 6.165 | 6.321 | <0.001 | 26.799 | 51.137 |
| age | 0.082 | 0.145 | 0.059 | 0.575 | -0.205 | 0.368 |
| sex | -26.88 | 8.526 | -3.153 | <0.005 | -43.709 | -10.051 |
| sex*age | 0.567 | 0.199 | 2.846 | <0.01 | 0.174 | 0.950 |
| ***c) regression analysis of temporal discrimination thresholds***  ***in healthy participants and unaffected relatives*** | | | | | | |
| ***interaction of group and age*** *R^2^=0.196, F(3,393)=31.758, p<0.001* | | | | | | |
|  | coefficient | SE | t value | p value | 95% confidence intervals | |
| intercept | 21.77 | 4.103 | 5.306 | <0.001 | 13.703 | 29.835 |
| age | 0.772 | 0.098 | 7.383 | <0.001 | 0.530 | 0.915 |
| group | 3.244 | 6.351 | 0.511 | 0.610 | -9.242 | 15.370 |
| Group*Age | -0.343 | 0.150 | -2.293 | <0.05 | -0.594 | -0.049 |
